# Quercetin: Its Main Pharmacological Activity and Potential Application in Clinical Medicine

**DOI:** 10.1155/2020/8825387

**Published:** 2020-12-30

**Authors:** Dengyu Yang, Tiancheng Wang, Miao Long, Peng Li

**Affiliations:** Key Laboratory of Zoonosis of Liaoning Province, College of Animal Science & Veterinary Medicine, Shenyang Agricultural University, Shenyang 110866, China

## Abstract

Quercetin is a flavonoid compound widely present in plants and exhibits a variety of biological activities. Research on quercetin has shown its potential for medical application. In this research, we elucidate its antioxidant mechanism and the broad-spectrum antibacterial and antiparasite properties; summarise its potential application in antioncology and cardiovascular protection and anti-immunosuppression treatment; and demonstrate its ability to alleviate the toxicity of mycotoxins. This research is expected to offer some insights and inspirations for the further study of quercetin, its properties, and the scientific basis for its better application in clinical practice.

## 1. Introduction

**Figure 1:**
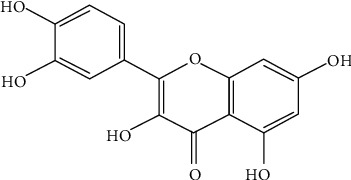
Structural formula of quercetin.

Quercetin, the name coming from quercetum (oak forest), named after Quercus, has been applied since 1857. It is widely found in plants in nature, including apples, berries, brassica vegetables, capers, grapes, onions, spring onions, tea, and tomatoes, as well as in many seeds, nuts, flowers, bark, and leaves [1]. However, quercetin is also contained in medicinal plants, including *Ginkgo biloba*, *Hypericum perforatum*, and elderberry [2–4], and is mainly derived from onions, apples, and tea [5]. Its molecular formula is C_15_H_10_O_7_, and the chemical structural formula is illustrated in Figure 1. It is a naturally occurring polar auxin transport inhibitor [6]. Quercetin has a ketocarbonyl group in its molecule, and the oxygen atom on the first carbon is basic and can generate salts with strong acids. Its molecular structure contains four active groups, namely, a dihydroxy group between the A ring, o-dihydroxy group B, C ring C2, C3 double bond, and 4-carbonyl. The presence of a phenolic hydroxyl group and double bonds endows quercetin with a strong antioxidant activity. Its antioxidant and anti-inflammatory properties are closely related to the prevention and treatment of cardiovascular diseases and cancer. In addition, *in vivo* and *in vitro* studies have found that quercetin also has antibacterial activity and effectively reduces the formation of biofilms by inhibiting the expression of related genes, antitumour activity, antiangiogenic activity, etc. In addition, quercetin plays an important role in reducing mycotoxins, protecting cells from damage. We have selected and analysed the key aspects of the biological functions of quercetin and its potential applications in clinical medicine to reach a unified understanding of its various functions. This review is designed to help with further research, and its nature is to provide some insights and enlightenment, providing a scientific basis for its better clinical application.

## 2. The Main Pharmacological Activity of Quercetin

### 2.1. Antioxidant

Free radicals are produced by the body during metabolism and are among the causes of many diseases. They can cause cell membrane damage and gene mutation, accelerate aging of the body, and induce various diseases, such as heart disease, liver damage, and diabetes [7, 8]. Hanasaki et al. [9] found that quercetin is the most effective free radical scavenger in the flavonoid family. By investigating the chemical structure of quercetin, it was found that there are four hydroxyl groups on the benzo-dihydropyran ring of the polyphenol, so quercetin has a strong antioxidant capacity, can eliminate free radicals produced in the body, and can help the body maintain a stable state.

The antioxidant mechanisms of quercetin *in vitro* mainly include the following:

1. Directly scavenging free radicals: Oh et al. [10] proved that quercetin had a strong antioxidant capacity, and it showed the highest antioxidant activity in all test samples. In addition, Manca et al. [11] found that quercetin adulterated with liposomes and glycerol nanoparticles could scavenge free radicals and protect human keratinocytes from hydrogen peroxide damage *in vitro*.
2. Chelating metal ions: related studies have confirmed that quercetin can induce Cu^2+^ and Fe^2+^ to play an antioxidant role through catechol in its structure. Tang et al. [12] fed adult male C57BL/6J mice to form a model of alcoholic liver disease and treated them with quercetin. The results suggested that quercetin could inhibit Fe^2+^-induced lipid peroxidation by binding Fe^2+^ and finally inhibit iron overload and oxidative damage in alcoholic liver disease. Babenkova et al. [13] undertook a chemiluminescence study to demonstrate that Fe^2+^ in compounds containing dihydroquercetin is inactive, unable to catalyse the decomposition of hydrogen peroxide, and unable to trigger further generation of hydroxyl free radicals. Therefore, quercetin can play the role of antioxidant stress through various cohorts and Fe^2+^.
3. Inhibiting lipid peroxidation: Lim et al. [14] confirmed that quercetin could inhibit the oxidative modification of low-density lipoprotein by observing the changes in the fluorescence intensity of thiobarbital, phosphatidylcholine hydroperoxides, and oxidised low-density lipoprotein, thus inhibiting the oxidative damage of LDL. Mbikay et al. [15] verified that, at low concentrations, quercetin can increase the expression of LDL-R, reduce the secretion of PCSK9, stimulate the uptake of LDL, and thus inhibit LDL oxidative damage.

The antioxidation mechanisms of quercetin *in vivo* are mainly such that the antioxidant capacity of quercetin is gradient-dependent and a high concentration of quercetin confers strong antioxidant capacity:

1. The antioxidant characteristics of quercetin: these are mainly manifested in the regulation of glutathione levels to enhance antioxidant capacity. When ROS are generated, SOD-2 will quickly capture O^2-^ and convert it into H_2_O_2_. GSH-Px catalyses the degradation of H_2_O_2_ to water molecules, which requires glutathione to provide reducing hydrogen [16].
2. Effects on enzyme activities: Odbayar et al. [17] found that quercetin can increase the expression of some antioxidant enzymes, such as glutathione transferase and aldo-keto reductase. The level of expression is proportional to the amount of quercetin.
3. Impact on signal transduction pathway: Wang et al. [18] showed that quercetin had a protective effect on granulosa cells by upregulating the expression of some genes related to oxidative stress *in vivo* and *in vitro*. In addition, Granado-Serrano et al. and Kobori et al. [19, 20] verified that quercetin upregulates the expression of Nrf2 and nuclear transfer by activating the intracellular p38 MAPK pathway, increasing the level of intracellular GSH, and affecting antioxidant enzyme activities, so that the antioxidant capacity of the cell is improved.

### 2.2. Antimicrobial Properties

Studies have shown that quercetin has broad-spectrum antibacterial properties; it not only has a good inhibitory effect on bacteria but also has a significant inhibitory activity on fungi. Several experiments have found that quercetin has a good inhibitory effect on the growth of pathogenic bacteria such as *Pseudomonas aeruginosa*, *Salmonella enteritidis*, *Staphylococcus aureus*, *Escherichia coli*, *Proteus*, and *Aspergillus flavus* [21, 22]. Hossion et al. [23] found that novel, artificially designed and synthesised, quercetin acyl glucosides effectively inhibited the growth of *E. coli*, *S. aureus*, and *P. aeruginosa.* In addition, bayberry extract has significant antibacterial activities against *Salmonella*, *Listeria*, and *Shigella* with the minimum inhibitory concentration (MIC) values ranging from 2.07 to 8.28 mg/mL [24].

According to current research, the antibacterial mechanism of quercetin mainly includes destroying the cell wall of bacteria and changing the cell permeability, affecting protein synthesis and expression, reducing enzyme activities, and inhibiting nucleic acid synthesis. Wang et al. [22] used TEM images to demonstrate that quercetin could damage the cell wall and membrane of *S. aureus* (at 10 × MIC) and demonstrated that treatment of *E. coli* (at 50 × MIC) with quercetin eventually led to cavitation and death. Zhao et al. [25] found that sugarcane bagasse (with 470 mg quercetin/g polyphenol) extract showed bacteriostatic activities against the growth of *S. aureus*, *L. monocytogenes*, *E. coli*, and *S. typhimurium*. In addition, Plaper et al. [26] found that quercetin altered the activity of ATP, thereby affecting the growth of *E. coli*. Wang et al. [27] found that quercetin can protect rats from catheter-related *S. aureus* infection by inhibiting thrombin activities. The relevant experiments on quercetin in inhibiting bacteria in recent years are summarised in Table 1.

In addition, quercetin can prevent bacterial adhesion, inhibit quorum sensing pathways, destroy or change the plasma membrane, inhibit efflux pumps, and block nucleic acid synthesis. Wang et al. [18] confirmed that quercetin inhibits the formation of *Streptococcus pneumoniae* biofilms. Qayyum et al. [36] found that quercetin was effective against *Enterococcus faecalis MTCC 2729* at the subminimal inhibitory concentration (sub-MIC), and scanning electron microscopy (SEM) and confocal laser scanning microscopy (CLSM) were used to elucidate that quercetin inhibited 95% of biofilm formation at 1/2 × MIC (256 g/mL). Kim et al. [37] found that a quercetin-pivaloxymethyl conjugate (Q-POM) at 5 *μ*g/mL inhibited 70% of biofilm establishment by a vancomycin-resistant *E. faecium* isolate. Vazquez-Armenta et al. [38] found that quercetin would hinder the abiotic surface colonisation of *Listeria monocytogenes* at concentrations below the MIC. In addition, Lee et al. [39] obtained that quercetin has an inhibitory effect on genes related to bacterial adhesion. Cho et al. [40] found that quercetin could significantly inhibit the production of biofilms of a methicillin-sensitive *S. aureus* strain (MSSA) ATCC 6538 after 24 h at concentrations of 20 *μ*g/mL and 50 *μ*g/mL.  Table 2 summarises the antibiofilm effect of quercetin on bacteria.

Quercetin has a broad inhibitory effect on bacteria, but as far as the current research on the fungal inhibitory effect is concerned, its fungal inhibitory effect is not as obvious as that on bacteria. Quercetin has no antifungal effect on *Clostridium neospora* when used alone, but when used together with AmB (amphotericin B), the antifungal activity is greatly improved. This implies that quercetin is a potential adjuvant drug for antifungal treatment of AmB [48]. Gao et al. found that quercetin is a beneficial antifungal drug in the clinical management of Candida vaginitis caused by *Candida albicans* biofilms and is a promising synergistic agent with fluconazole [49]. Quercetin enhances fluconazole-resistant *Candida albicans*-induced apoptosis by regulating quorum sensing [50]. The relevant experiments of the inhibitory effect of quercetin on fungi in recent years are summarised in Table 3.

## 3. Applications of Quercetin

### 3.1. Antitumour

Many studies have shown that quercetin can exert antitumour effects through various mechanisms, which has been confirmed in various tumour *in vivo* and *in vitro* models. Quercetin can significantly prevent the cell cycle, promote cell apoptosis, and inhibit blood vessel generation and transfer. Lee et al. [56] found that in human leukaemia U937 cells, quercetin induces cell cycle arrest at G2 (late DNA synthesis phase). Suh et al. [57] found that quercetin can also induce G0/G1 (pre-DNA synthesis) phase changes in 232B4 chronic lymphocytic leukaemia cells and HOS osteosarcoma cells. In addition, Chou et al. [58] have proved that quercetin also affects the regulation of p53-related pathways in the tumour cell cycle. Their experiments discovered that quercetin can induce ER stress and promote the release of p53, thereby inhibiting the activities of CDK2, cyclin A, and cyclin B, thereby causing MCF-7 breast cancer cells to stagnate in the S phase. In addition, Hamidullah et al. [59] found that in PC-3 and DU145 prostate cancer cell lines, a certain dose of quercetin-6-C-*β*-D-glucopyranoside treatment can lead to cell cycle arrest in the G0/G1 phase. This phenomenon may be related to the downregulation of cyclins E and D, PNCA, and Cdk-2 protein expression and increased expressions of p21 and p27 (Table 4).

Quercetin can affect the cancer cell apoptosis pathway and induce tumour cell death. Experiments have shown that a reasonable dose of quercetin can increase the expression of proapoptotic protein and reduce the expression level of antiapoptotic protein. Granato et al. [60] found that quercetin inhibited the PI3K/AKT/mTOR and STAT3 pathways in PEL, which downregulated the expression of survival cell proteins such as c-FLIP, cyclin D1, and cMyc. Deng et al. [61] found that quercetin induced MCF-7 cell apoptosis and inhibited the proliferation of MCF-7 breast cancer cells in a time and concentration-dependent manner, thereby inhibiting breast cancer cells. In addition, Teekaraman and others [62] studied the role of quercetin apoptosis in the human metastatic ovarian cancer PA-1 cell line. The results showed that quercetin induced the mitochondrial-mediated apoptosis pathway, thereby inhibiting metastatic ovarian cancer cell growth. Seo et al. [63] showed through experiments that quercetin induced apoptosis at concentrations in excess of 20 *μ*M by inhibiting STAT3 signalling and could be used as a useful compound for the prevention or treatment of breast cancer overexpressing HER2 (Table 5).

With the development of clinical trials, the great potential of quercetin in the treatment of cancer has been further confirmed; however, there remain some limitations in the scope and number of clinical trials involved, and more comprehensive clinical trials are needed to confirm its therapeutic effect on tumours.

### 3.2. Anti-Inflammatory and Immunosuppressive Effects

Quercetin has been confirmed to be a long-acting anti-inflammatory substance in flavonoids [64, 65]. Both in animal and in human models, quercetin can show significant anti-inflammatory potential in different cell types [66, 67]. The plant extract of quercetin is used as the main component of many potential antiallergic drugs. Compared with Cromolin (the antiallergic drug disodium cromoglycate), its ability to inhibit IL-8 is stronger and can inhibit IL-6 and increase cytosolic calcium levels [68]. Its anti-inflammatory and antiallergic properties have been validated in the treatment of respiratory and food allergies [69, 70]. In addition to a wide range of biochemical and pharmacological activities, quercetin has been repeatedly shown to exert anti-inflammatory effects on endothelial and monocyte/macrophage systems *in vitro* [71, 72].

Li et al. [73] conducted experiments in different animal models and found that quercetin inhibited the production of tumour necrosis factor alpha (TNF-*α*) induced by lipopolysaccharide (LPS) in macrophages [66] and lung A549 cells LPS-induced IL-8 production [67]. Furthermore, it has even been shown in glia cells that quercetin can suppress LPS-induced mRNA levels of TNF-*α* and interleukin- (IL-) 1*α*: neuronal cell death is also reduced [74]. Quercetin can inhibit the enzymes that produce inflammation (cyclooxygenase (COX) and lipoxygenase (LOX)) [75].

According to several studies on the correlation between quercetin and its immunomodulatory effects, quercetin can reduce disease after strenuous exercise. Nieman et al. showed that, among well-trained cyclists, supplementing quercetin and epigallocatechin-3-gallate (Q-EGCG) for two weeks could enhance GOBA granulocytes and resist inflammation after three days of heavy exercise [75]. In addition, in clinical trials, quercetin and resveratrol, EGCG, and genistein have been found to enhance cellular and humoral immune functions [76].

### 3.3. Cardiovascular Protection

The quercetin exerts beneficial effects on cardiovascular diseases, such as hypertension, atherosclerosis, ischemia-reperfusion injury, or cardiotoxicity [77–79], which are closely associated with the anti-inflammatory and antioxidant properties of quercetin. The protective mechanism of quercetin on the cardiovascular system includes (1) reducing systolic blood pressure, diastolic blood pressure, and mean arterial pressure. (2) The levels of ST segment, lipid peroxidation in the plasma and heart, free fatty acid, phospholipid, total cholesterol, and triglyceride in serum were decreased. (3) It can regenerate blood vessels and reduce blood sugar. (4) It can effectively decrease the thickness of the aortic wall. Edwards et al. [80] found that, among patients with stage 1 hypertension, those who took 730 mg of quercetin for 28 days had a decrease in their systolic, diastolic, and mean arterial pressure. Quercetin presents significant heart-inhibiting effects on LDL oxidation and endothelium-dependent vasodilation [81] and reduces the effects of adhesion molecules and other inflammation markers. In addition, a study of 93 overweight or obese subjects at high risk of metabolic syndrome who were given a daily dose of 150 mg quercetin for six weeks showed significant reductions in plasma concentrations of LDL oxidised by systolic blood pressure and atherosclerosis [82]. The protective effect refers to the effects of nitrogen oxide (NO) and endothelial function and the prevention of oxidative inflammatory damage of neurons and the antiaggregation effect of platelets. Wei et al. [83] found that quercetin has a potential for use in treating heart disease as quercetin treatment is found to be capable of reducing LPS-induced cardiac abnormalities in mice.

Quercetin can control dyslipidaemia, and changes in fatty liver functions are essential for controlling serum fat levels. Gnoni et al. investigated the effect of quercetin on rat hepatocyte fat production [84]. The experiment found that the addition of quercetin to liver cells at a concentration of 25 *μ*M, within 30 minutes could inhibit the synthesis of fatty acids. Tian et al. [85] found that 50 *μ*M 7-O-sialic acid (QA) can protect human umbilical vein endothelial cells. In addition, study showed that quercetin (10 mg/kg) orally administered to rats for seven consecutive days protected them from experimental myocardial infarction [86]. Kleemann et al. [87] demonstrated that quercetin could downregulate the expression of C-reactive protein and cardiovascular risk factors (SAA, fibrinogen) in mice. These results indicated that quercetin might have cardiovascular protective effects.

Quercetin also protected mice fed a high-fat diet from endothelial dysfunction caused by oxidants and protected apolipoprotein E-knockout mice from atherosclerosis [88]. Some studies have shown that quercetin positively influences the development of the embryo, foetus, and placenta. Since this flavonoid has no teratogenic and miscarriage effects, it is generally considered safe. Therefore, in this risk group, its potential use in the prevention and treatment of pregnancy-induced hypertension syndrome has received much research attention [89–91].

### 3.4. Quercetin Relieves Mycotoxin Toxicity

According to multiple studies, quercetin can alleviate the toxicity of mycotoxins. Quercetin alleviates mycotoxin toxicity due to its antioxidant and anti-inflammatory properties. Quercetin alleviates mycotoxins by protecting cells from endoplasmic reticulum stress and apoptosis induced by mycotoxins, increasing the level of glutathione peroxidase, enhancing the activity of oxide dismutase, increasing the activity of catalase, reducing the lipid peroxidation reaction, and decreasing the level of ROS (Table 6). Ben et al. [92] found that the antioxidant activity of quercetin and saffron can decrease the level of ROS produced by ZEN, inhibit ER stress, and protect HCT116 and HEK293 cells from ZEN-induced apoptosis. Further research by Ben et al. [93] proved that quercetin could prevent a/b-ZOL-induced ROS generation in HCT116, prevent a-ZOL and b-ZOL-induced ER stress, and reduce a-ZOL and b-ZOL-induced apoptosis. Their experiments show that quercetin protects HCT116 cells from a-ZOL and b-ZOL-induced apoptotic cell death. This is in good agreement with the existing literature on quercetin as an antioxidant in various types of oxidative damage [94, 95].

Aflatoxin B1 (AFB1) is a common mycotoxin found in feed, which has a variety of toxic effects. The neurotoxicity of AFB1 can lead to memory disorder. Quercetin plays a preventive role in antioxidant stress by promoting the antioxidant defence system and limiting lipid peroxidation. Studies have shown that quercetin can increase the level of glutathione peroxidase (GSH) and the activity of superoxide dismutase (SOD) and catalase (CAT) in the brain and reduce the lipid peroxidation of AFB1-treated mice. This is consistent with the effect of quercetin on behavioural and cognitive impairment in a Parkinson's disease model [96] and a chronic cerebral ischemia model [97]. Quercetin can significantly reduce the synthesis of AFB1. In recent years, it has been found that quercetin in tea polyphenols can hinder the conversion of aflatoxin AFB1 to the carcinogenic product AFB1-8,9-epoxide [98], which matches the findings in a study by Ghadiri et al. [99]. Resveratrol and quercetin (both 5 *μ*M) (to a lesser extent) significantly offset the impaired cell viability mediated by AFB1 (concentration range: 96-750 *μ*M). There are toxicological implications associated with AFB1 intake such as hepatotoxicity and carcinogenicity. Quercetin can detoxify AFB1 by regulating the activity of glutathione and SOD; also, the participation of mitochondria and lysosomes in AFB1-induced cytotoxicity might be a possible proposed mechanism thereof.

Quercetin pretreatment can inhibit aflatoxin-induced cytotoxicity and oxidative stress, mainly by activating Nrf2 pathway to regulate changes to the antioxidant defence system induced by Aspergillus. In addition, quercetin also shows antigenic toxicity potential by reducing DNA damage and micronucleus (MN) damage induced by the Aspergillus toxin. Ramyaa et al. [100, 101] first found that quercetin pretreatment can inhibit ochratoxin-induced cytotoxicity and oxidative stress. Schoneberg et al. [102] found that the contents of NO, TNF-*α*, IL-6, and IL-8 of ochratoxin were significantly reduced in samples pretreated with quercetin, indicating that quercetin had anti-inflammatory effects. It has been proved that quercetin has a cytoprotective effect on ochratoxin-induced oxidative stress, genotoxicity, and lymphocyte inflammation [103]. Bollina and Kushalappa [104] found that the addition of quercetin at a concentration of 2.95 mM reduced the production of deoxynivalenol (DON) by *Fusarium graminearum in vitro*, but no obvious concentration response was found in mycotoxins. The protective effects of quercetin on key mycotoxin toxicities and their mechanism are summarised in Table 6.

### 3.5. Other Functions

Currently, quercetin extract is widely used as a nutritional supplement and therapeutic ingredient for many diseases, such as diabetes, which is associated with obesity and circulatory dysfunction (including inflammation and emotional distress) [115]. Previous experiments showed that quercetin can inhibit fat production and benefit obese people [116]. The mechanism of action of quercetin is pleiotropic, involving inhibition of intestinal glucose absorption, insulin secretion, and insulin sensitisation activities, and improvement of glucose utilisation in peripheral tissues [78]. In addition, quercetin helps reduce lipid peroxidation, platelet aggregation, and capillary permeability and may be used in the treatment of obesity and type 2 diabetes [117, 118]. Quercetin also reduces obesity-induced skeletal muscle atrophy by inhibiting inflammatory receptors and their signalling pathways. Quercetin is used to prevent obesity-induced muscle inflammation and sarcopenia [119]. Ying et al. [120] suggested that quercetin can decrease the levels of malondialdehyde (MDA) and NO by changing the activity of antioxidant enzymes, activating the expression of PI3K/PKB signalling pathway-related genes, regulates glucose metabolism, reduces oxidative damage, and has a protective effect on ascorbic acid therapy.

Quercetin has been shown to be important in the fight against parasites and has been demonstrated in different clinical trials, such as those against *Leishmania*, *Trypanosoma*, and *Plasmodium*. The antiparasitic effect is related to the destruction of mitochondrial function and the inhibition of different important enzymes and molecules, including heat-shock protein (HSP), acetylcholinesterase, DNA topoisomerase, and kinase (Table 7).

In addition, quercetin can reverse cognitive impairment and enhance memory in the ageing process. Quercetin has the protective effects of antioxidant damage and neuroinflammation, so it is a potential therapeutic candidate for the treatment of neurological diseases and is helpful for the treatment of cognitive impairment [126, 127]. Multiple experiments have shown that quercetin has a neuroprotective effect [128]. Ishisaka et al. and Das et al. [129, 130] found that rodents can be protected from various forms of neurotoxic damage after oral administration of quercetin (0.5-50 mg/kg). Quercetin can also protect nerve damage caused by heavy metals, such as lead and mercury [131–133]. In addition, quercetin can also reduce nerve damage caused by chemicals, such as the insecticide endosulfan [134, 135].

## 4. Summary and Future Prospects

Quercetin has shown good therapeutic activities against various diseases. Through continuous research, quercetin is expected to become a new drug that can prevent and treat various diseases. Its powerful antioxidant, anti-inflammatory, and antitumour effects have great prospects in clinical application. At this stage, the antioxidants added to animal feed have carcinogenic, teratogenic, mutagenic, and other side effects on humans and animals. Quercetin is a safe, natural antioxidant and can be used in animal feed. At the same time, when quercetin exerts antioxidant activities in the body, it can also improve physical functions and reduce stress reactions. The author believes that the level and effect of quercetin in different animal feeds need further in-depth discussion.

The broad-spectrum antimicrobial properties of quercetin can be used in the prevention and treatment of various infectious bacterial diseases and can provide treatment options to reduce the use of antibiotics, which has important implications for the safety and sustainable development of human and animal health: however, at present, research into the antibacterial effect of quercetin is mainly focused on the antibacterial activity of quercetin, but there is little research on the antifungal effect. Whether the antibacterial mechanism of quercetin is akin to those of fungi and bacteria or whether it has inhibitory effects on different types of fungi still needs further experimental research.

According to the broad-spectrum antimicrobial properties, application as a preservative is expected. In addition, quercetin antioxidant treatment may help to prevent mycotoxin toxicity in food and feed industry. However, in terms of the present study, the absorption of quercetin in the human body and the metabolic mechanism are not clear. Further research into quercetin is needed before pharmacological application.

## Figures and Tables

**Table 1:** The inhibitory effect of quercetin on bacteria.

Bacteria	Whether it has inhibitory effect	Mechanism
Aerobic bacteria [28]	Yes	Inhibits nucleic acid synthesis and destroys cell plasma membrane and energy metabolism

E. coli [22, 28, 29]	Yes	Inhibits nucleic acid synthesis and disrupts plasma membrane function

Pseudomonas aeruginosa [22, 28]	Yes	Inhibits nucleic acid synthesis and disrupts plasma membrane function

Salmonella typhimurium [22]	Yes	Inhibits nucleic acid synthesis and disrupts plasma membrane function

Staphylococcus aureus [22]	Yes	Inhibits nucleic acid synthesis and disrupts plasma membrane function

Drug-resistant E. coli [30]	Yes	By destroying bacterial cell walls and cell membranes

Bacillus subtilis [30]	Yes	By destroying bacterial cell walls and cell membranes

Enterococcus faecalis [31]	Yes	Inhibits the synthesis of Enterococcus faecalis naphthalate synthase

Mycobacterium [32]	Yes	Quercetin-3-O-*β*-D-glucoside inhibits glutamine synthetase

Aerobes [29]	Yes	Inducing antibacterial effects by inhibiting the supercoiled activity and DNA cleavage of bacterial gyrase

Bifidobacterium [29]	No	\

Lactobacillus [29]	No	\

Streptococcus mutans [33]	Yes	Reduces dry weight of biofilm and total protein

Carbapenem-resistant Pseudomonas aeruginosa [34]	Yes	Destroys cell wall integrity and changes cell morphology to exert bactericidal activity

Acinetobacter baumannii [34]	Yes	Destroys cell wall integrity and changes cell morphology to exert bactericidal activity

Carbapenem-resistant Pseudomonas aeruginosa [35]	Yes	Through alteration of blaVIM, ompC expression, and cellular morphology of bacteria

Klebsiella pneumoniae [35]	Yes	Through alteration of blaVIM, ompC expression, and cellular morphology of bacteria

**Table 2:** Antibiofilm effect of quercetin on bacteria.

Biofilm-producing strains	Quercetin/quercetin conjugate	Effect
*Bacillus subtilis strain FB17* [41]	Quercetin	Quercetin diminished biofilm formation

*Enterococcus faecalis MTCC 2729* [36]	Quercetin	At submic concentrations, quercetin inhibits biofilm formation. Compared with the control group, 10 and 9 proteins were overexpressed and effective after quercetin treatment

A vancomycin-resistant *Enterococcus Faecalis* [37]	Quercetin–pivaloxymethyl conjugate (Q-POM)	Q-POM efficiently hampered biofilm formation in a dose-dependent manner

*Staphylococcus aureus ATCC 6538* [39]	Quercetin	Quercetin not only abolished the biofilm forming and hemolytic S. aura but also suppresses the expression of adhesion-related, quorum sensing, and virus-regulatory genes

*Staphylococcus aureus ATCC 25923* [39]	Quercetin	Quercetin not only abolished the biofilm forming and hemolytic S. aura but also suppresses the expression of adhesion-related, quorum sensing, and virus-regulatory genes

*A clinical isolate of Staphylococcus aureus* [42]	Quercetin-AgNP hybrid	Quercetin-AgNP hybrid significantly reduced the formation of biofilms and the production of extracellular polymers

*MSSA ATCC 29213*, *MRSA ATCC 33591*, and clinical isolates of *Staphylococcus aureus* [43]	Quercetin	Quercetin (at MIC and sub-MICs) inhibited around 50% of biofilm establishment

*Streptococcus mutans strain Ingbritt* [44]	Quercetin-doped adhesive groups	Compared with the control group, the binder group doped with quercetin showed antibacterial activity, acceptable biocompatibility, inhibition of matrix metalloproteinases, and an effective bonding interfacial seal

Clinical isolates of *Pseudomonas Aeruginosa* [45]	Quercetin	Quercetin basically inhibits biofilm formation and twitching movements

*Proteus mirabilis HI4320* [46]	Quercetin	Quercetin dose dependently increased biofilm establishment

*Streptococcus pneumoniae strain D39* [47]	Quercetin	Quercetin reduced biofilm formation and CFUs in a dose-dependent manner

**Table 3:** The inhibitory activity of quercetin against fungi.

Fungi	Synergistic effect of quercetin	MIC
*Aspergillus flavus* [51]	NO	505 *μ*g/mL
*Candida tropicalis resistant to fluconazole* [52]	Fluconazole	128 *μ*g/mL of flavonoids, combined with fluconazole (16 *μ*g/mL)
*Actinobacillus actinomycetemcomitans (Aa)* [53]	NO	0.1 g/mL
*Porphyromonas gingivalis (Pg)* [53]	NO	0.0125 g/mL
*Candida albicans* [53]	NO	No effect
*Rhizopus azygosporus* [54]	NO	No effect
*Candida parapsilosis* [55]	NO	0.5 *μ*g/mL
*Cryptococcus neoformans ATCC 90012* [48]	Amphotericin B	0.125 *μ*g/mL

**Table 4:** Inhibitory effect of quercetin on different cancer cells.

Cancer type	Cell lines	Cell cycle	Mechanism
Lymphoma [56]	U937	G2/M	↑ Cyclin B
Osteosarcoma [57]	HOS	G1/S	↓ Cyclin D1
Breast cancer [58]	MCF-7	S	↓ CDK2, cyclins A and B ↑ p53, p58
Prostate cancer [59]	PC-3, DU-145	G0/G1	↓ Cyclins E and D, PNCA, Cdk-2 ↑ p21, p27

**Table 5:** Effects of quercetin on apoptosis of different tumour cells and its mechanism.

Cancer type	Mechanism	Signalling pathway
Lymphoma cell [60]	↓ c-FLIP, cyclin D1, cMyc	PI3K/AKT/mTOR/STAT3
Ovarian cancer [62]	↓ Cyclin D1, DNA-PK, phosphohistone H3 ↑ p21	—
Breast cancer [63]	↑ Caspase-3-8, p53, p21	STAT3
Lung cancer [27]	↓ Survivin ↑DR5	AKT-survivin

**Table 6:** Protect effects of quercetin on some main mycotoxin toxicity and its mechanism.

Mycotoxin	Mechanism	Effect
ZEN [92]	Antioxidant activity, ROS production ↓, ER256 ↓	Protecting HCT116 and HEK293 cells and inhibit cell apoptosis

a/b-ZOL [93]	ROS production ↓, inhibit a-zol, b-zol endoplasmic reticulum stress	Protecting cells from damage

AFB1 [98]	Reversing the negative regulation of GSTA1, increase GSH level ↑	Inhibiting AFB1 biotransformation

AFB1 [105]	↑ Increased the level of glutathione peroxidase, increase the activity of oxide dismutase, increased the activity of catalase, and ↓ reduced the lipid peroxidation reaction	Improved brain cognition and spatial memory, increased anxiety and drowsiness disorders

AFB1 [106]	↓ Reduced ROS generation, ↑ antioxidant enzyme activity	Improved the learning and memory impairment of mice

AFB1 [107]	Cross the blood-brain barrier	Quercetin could be a potential neuroprotective approach to slow degenerative disease progression

Ochratoxin A [108]	/	Protecting cells from damage

Deoxynivalenol cytotoxicity [104]	/	Protecting intestinal caco-2 cells from damage

AFB1 [109–112]	Inhibited CYP1A-mediated 7-ethoxyresorufin O-deethylase (EROD) activity in liver microsomes	Affects AFB1 biotransformation remains

Citrinin (CTN), patulin (PAT), and zearalenol (ZEAR) [113]	↓ Decreased cell viability and ↑ increased LDH activity	Protecting the cell lines from cytotoxicity

AFB1 [114]	↓ Decreasing the rate of ROS formation, lipid peroxidation and improved cell viability, mitochondrial membrane potential and glutathione level and reducing levels of aspartate aminotransferase, alanine aminotransferase, and alkaline phosphatase	Hepatoprotective effect

**Table 7:** Inhibitory mechanism of quercetin on several parasites.

Parasite	Mechanism of action
*Leishmania donovani* [121]	Low selectivity to parasite DNase I
*Trypanosoma brucei* [122]	Cause a loss of mitochondrial membrane potential and marked DNA degradation
*Plasmodium falciparum* [123]	Antiplasmodial potential
*Encephalitozoon intestinalis* [124]	Antiparasitic activity
*Leishmania mexicana* [125]	Inhibition of parasite cathepsin L

## Data Availability

The data used to support the findings of this study are included within the article.
